# Current Tobacco Smoking Prevalence Among Iranian Population: A Closer Look at the STEPS Surveys

**DOI:** 10.3389/fpubh.2020.571062

**Published:** 2020-12-16

**Authors:** Mohammad-Reza Sohrabi, Mohsen Abbasi-Kangevari, Ali-Asghar Kolahi

**Affiliations:** Social Determinants of Health Research Center, Shahid Beheshti University of Medical Sciences, Tehran, Iran

**Keywords:** epidemiology, passive smoking, public health, smoking, tobacco

## Abstract

**Objectives:** To determine the current tobacco smoking prevalence among Iranian adults, its geographical distribution in 2011, 2016, and time trend during 2004-2016.

**Methods:** We conducted a pooled analysis of the published reports of 2004, 2007, 2008, 2009, and the data of 2011 and 2016 of the STEPwise approach to chronic disease risk-factor surveillance (STEPS) surveys.

**Results:** The prevalence of current tobacco smoking and current daily cigarette smoking in 2016 was 14.1 and 9.7%, respectively. Only 0.2% of participants smoked water-pipe. Current tobacco smoking prevalence remained unchanged during 2004-2016 for both men and women. The prevalence of passive smoking at home or workplace was 27.4%. Current tobacco smoking prevalence and current daily cigarette smoking was significantly lower among women than men. Current tobacco smoking prevalence showed a geographical pattern throughout the country. In both 2011 and 2016, current tobacco smoking prevalence was higher among men who lived in the western provinces, especially the north-west, than those who lived in the eastern and southern provinces.

**Conclusions:** The current tobacco smoking prevalence among Iranian population has not changed significantly during 2004-2016 and does not conform to the international guidelines. Therefore, it remains crucial yet challenging that effective nationwide policies be implemented to reduce the use of tobacco products. One cannot hope for any reductions in smoking prevalence until a cocktail of interventions are built around strong commitment to government policy.

## Introduction

Smoking is the second leading risk-factor for deaths and disability-adjusted life-years (DALYs). It accounted for 7.6 million deaths in 2019, 13.6% of all deaths globally. In addition, smoking was considered responsible for nearly 200 million DALYs, 7.9% of all DALYs globally (1). Although smoking is a risk-factor for six of the eight leading causes of death (2), prevention of smoking as a modifiable risk-factor has received improper attention. Therefore, the 2030-Agenda-for-Sustainable-Development-Goals has highlighted the necessity for a joint-action to reduce current-smoking-prevalence (3).

Previous efforts to reduce the smoking prevalence have resulted in a 1.6% decline per-year from 1990 to 2015 worldwide (4). However, World Health Organization (WHO) has called for a 5.8% decline per-year and exceeds the achievements of countries in recent years (5). If the current trends persist worldwide, it could be assumed that the total number of annual deaths increase by ten million by 2030 (6). Thus, the effectiveness of previous efforts to reduce smoking prevalence needs to be constantly monitored via surveillance programs to take the required public health measures promptly.

Surveillance programs, such as STEPwise approach to chronic-disease-risk-factor-surveillance (STEPS) developed by WHO and demographic health survey, are being implemented by countries which focus on obtaining data on established risk-factors that determine the burden of major non-communicable-diseases. Iran has implemented and conducted the STEPS survey six times. The first STEPS survey in Iran was conducted in 2004, and the latest was in 2016.

The prevalence of current tobacco smoking, time trends and geographical distribution among the Iranian population needs to be determined as a step toward taking the necessary nationwide measures for proper prioritization of risk-prevention programs to address the issue.

The objective of this study was to determine the prevalence of current tobacco smoking in 2016, its geographical distribution by province in 2011 and 2016. In addition, we investigated the time trend of current tobacco smoking prevalence during 2004-2016 via a pooled analysis of published reports of national STEPS surveys.

## Methods

The study was approved by Ethical Committee of Shahid Beheshti University of Medical Sciences under the reference code IR.SBMU.RETECH.REC.1397.068.

### Data Source

All available data and reports of the STEPS studies in Iran were used in the study, which were nationwide cross-sectional surveys of surveillance of risk-factors of non-communicable diseases. So far, STEPS has been conducted six times in Iran: 2004, 2007, 2008, 2009, 2011, and 2016. We had access to the raw data of SETPS 2011 and 2016. However, the data of STEPS 2004, 2007, 2008, 2009 were not available and the published reports in the website of WHO and National Institute of Health of Iran were used (7). The surveys were conducted on individuals more than 18 years living in urban and rural areas of Iran through systematic-proportional-to-size-cluster-sampling. Detailed description of study population and the sampling method of the 2016 version of the STEPS survey has been published elsewhere (8, 9). The data of all the 31 provinces of Iran were collected in all studies, except for Qom Province in 2016.

### Variables

Smoking was assessed using the transcultural-adaption of STEPS-questionnaire in national STEPS surveys. Variables included sociodemographic status of participants including age, sex, place of residence, occupation, and smoking-related variables. Smoking-related variables of STEPS 2016 included forms of smoking including cigarette smoking and water-pipe use; amount of cigarette smoking; onset age of smoking; history of attempts to quit smoking; history of passive smoking; place of passive smoking; and whether a healthcare professional had advised against smoking. We assessed current tobacco smoking for all STEPS surveys, and current daily cigarette smoking and passive smoking for STEPS 2016. Current tobacco smoking was defined as the use of any smoked tobacco products, including cigarettes, cigars, pipes, or any other smoked tobacco products, on a daily, non-daily or occasional basis. The main tobacco-containing products used by Iranians are cigarette and water-pipe, while chewing tobacco and other forms are uncommon. Current daily cigarette smoking was defined as smoking any amounts of cigarettes daily. Passive smoking was defined as being exposed to the smoke of any tobacco products in the past 30 days.

### Statistical Analysis

Frequency, proportion, mean, and standard deviation (SD) were used to describe the data. We used chi-square test for categorized variables. *T*-test and one-way analysis of variance (ANOVA) test were used to analyze the differences among means of two groups and three groups or more, respectively. The latter tests were performed using IBM SPSS Statistics version 21. Exploratory Data Analysis were performed to investigate the correlation between current tobacco smoking in 2016 among provinces and socioeconomic variables. The Exploratory Data Analysis was performed in and the figures were designed by Tableau Desktop 2018 (Washington, USA).

## Results

The total number of participants was 89,404 in 2004, 29,994 in 2007, 29,775 in 2008, 29,888 in 2009, 12,104 in 2011, and 30,541 in 2016 (Table 1).

**Table 1:** Socio-demographic status of participants from 2004 to 2016.

**Variables**	**2004** ***n* = 89,404**	**2007** ***n* = 29,994**	**2008** ***n* = 29,775**	**2009** ***n* = 29,888**	**2011** ***n* = 12,104**	**2016** ***n* = 30,541**
Age, mean (SD)	39.5 (14.3)	39.5 (14.2)	39.5 (14.3)	39.5 (14.2)	37.2 (17.9)	44.5 (16.3)
**Residential area**, ***n*** **(%)**						
Urban	57,794 (64.6)	17,698 (59.1)	18,419 (61.8)	16,239 (54.3)	8,343 (68.9)	21,493 (70.4)
Rural	31,610 (35.4)	12,244 (40.8)	11,355 (38.1)	13,648 (45.6)	3,761 (31.1)	9,048 (29.6)
**Sex**, ***n*** **(%)**						
Female	44,322 (49.6)	15,007 (50.1)	14,842 (49.8)	14,948 (50.1)	6,962 (57.5)	15,975 (52.3)
Male	45,082 (50.4)	14,987 (49.9)	14,933 (50.2)	14,940 (49.9)	5,142 (42.5)	14,566 (47.7)
**Employment status of women**						
Housewife	N/A^*^	11,991 (79.9)	11,458 (77.2)	11,764 (78.7)	5,389 (77.4)	12,967 (81.1)
Student	N/A	1,231 (8.2)	1,440 (9.7)	1,435 (9.6)	620 (8.9)	622 (3.9)
Employed	N/A	1,110 (7.4)	1,009 (6.8)	853 (5.7)	529 (7.6)	1,467 (9.2)
Other	N/A	675 (4.5)	935 (6.3)	896 (6.0)	424 (6.1)	919 (5.8)
**Employment status of men**						
Non-government employee	N/A	8,318 (55.5)	7,945 (53.2)	8,396 (56.2)	2,720 (52.9)	8,725 (59.9)
Government employee	N/A	1,978 (13.2)	1,971 (13.2)	1,509 (10.1)	411 (8.0)	1,544 (10.6)
Student	N/A	1,768 (11.8)	1,552 (10.5)	1,912 (12.8)	782 (15.2)	641 (4.4)
Other	N/A	2,923 (19.5)	3,465 (23.1)	3,123 (20.9)	1,229 (23.9)	3,656 (25.1)

* *Not available*.

### Smoking in 2016

#### Prevalence of Current Tobacco Smoking

The current tobacco smoking prevalence in 2016 was 4316 (14.1%). The current tobacco smoking prevalence was significantly lower among women than men: 643 (4.0%) vs. 3676 (25.2%), *P* < 0.001. The current tobacco smoking prevalence increased with age, from 13.5% among men aged 18–25 to the observed peak of 33.1% among men aged 51–55 years; however, it then decreased to 16.7% among men aged more than 70. Among women, the current tobacco smoking prevalence increased with age, from 2.0% among those aged 18–25 to 5.8% among those aged more than 80.

The current tobacco smoking prevalence was significantly lower among government employees than those who were non-government employees: 240 (16.1%) vs. 2473 (29.1%), respectively, *P* < 0.001. The current tobacco smoking prevalence among male students was 58 (9.3%) which was one-third of that of non-governmental employees. No significant differences were observed in the current tobacco smoking prevalence among women with various occupations. The mean (SD) onset age of smoking was significantly lower among men than women: 21.4 (7.9); 95% CI 20.9–21.8 vs. 27.8 (13.5); 95% CI 24.4–31.2, *P* < 0.001. The current tobacco smoking prevalence among participants was higher in rural than urban areas: 1370 (15.1%) vs. 2846 (13.2%); however, the difference was not statistically significant. There were no significant correlations between current tobacco smoking in 2016 among provinces and socioeconomic variables including age, female/male ratio, urban/rural ratio, employed/unemployed ratio.

#### Prevalence of Current Daily Cigarette Smoking

The prevalence of current daily cigarette smoking in 2016 was 2962 (9.7%). The prevalence of current daily cigarette smoking was significantly lower among women than men: 144 (0.9%) vs. 2849 (19.6%), *P* < 0.001. The mean (SD) number of daily smoked cigarettes was 14.6(9.5) among men and 13.1(10.1) women which was not different.

#### Attempt or Being Advised to Quit Smoking

Among all participants who smoked tobacco, only 607 (14.4%) had attempted to quit smoking during the previous year. The prevalence of participants who had attempted to quit smoking during the previous year was significantly higher among men than women: 538 (25.5%) vs. 69 (12.4%), *P* < 0.001. There was no difference between the urban and rural areas in attempting to quit smoking.

Among all participants who smoked, 803 (35.5%) had been advised to quit smoking by a healthcare professional during the previous year. The prevalence of participants who had been advised to quit smoking during the previous year was significantly higher among men than women: 709 (39.8%) vs. 94 (19.5%), *P* < 0.001. Smokers who had been advised to quit smoking were more likely to attempt quitting smoking compared with those who hadn't: 311 (38.8%) vs. 197 (13.5%), *P* < 0.001. Among those who attempted to quit smoking, there was no difference between the urban and rural areas in being advised to quit smoking by a healthcare professional.

#### Prevalence of Water-Pipe Smoking and Other Types of Tobacco

Only 51 (0.2%) participants, 29 (0.3%) men, and 22 (0.1%) women, smoked water-pipe. No participant used tobacco in its chewing form.

#### Passive Smoking

Among all participants, the prevalence of passive smoking was 9698 (31.8%): 5237 (36%) among men and 4461 (27.9%) among women. The prevalence of passive smoking among non-smoking participants was 7065 (27.4%). The prevalence of passive smoking among women was higher than men: 3048 (28.5%) vs. 4017 (26.6%); however, the different was not statistically significant. The prevalence of passive smoking among women and men is presented in Table 2. The prevalence of passive smoking was significantly higher in rural than urban areas: 2662 (35.5%) vs. 4403 (24.1%), *P* < 0.001. While women were more likely to be passive smokers at home, men were more likely to be passive smokers at work. The proportion of passive smokers at work was higher in urban than rural areas (Table 3).

**Table 2:** Comparison of the prevalence of passive smoking among men and women in 2016.

**Place**	**Women, *n* (%)**	**Men, *n* (%)**	***P*-value**
Only home	2,899 (72.2)	1,296 (42.5)	<0.001
Only work	237 (5.9)	965 (31.6)	<0.001
Both	881 (21.9)	788 (25.9)	NS^*^
Total	4,017 (100)	3,049 (100)	

* *Non-significant*.

**Table 3:** Comparison of the prevalence of passive smoking among participants in urban and rural areas in 2016.

**Place**	**Urban, *n* (%)**	**Rural, *n* (%)**	***P*-value**
Only home	2,541 (57.7)	1,654 (62.1)	NS^*^
Only work	937 (21.3)	265 (9.9)	<0.001
Both	925 (21.0)	744 (28.0)	<0.001
Total	4,403 (100)	2,663 (100)	

* *Non-significant*.

### Geographical Pattern of Smoking Prevalence in 2011 and 2016

**Figure 1:**
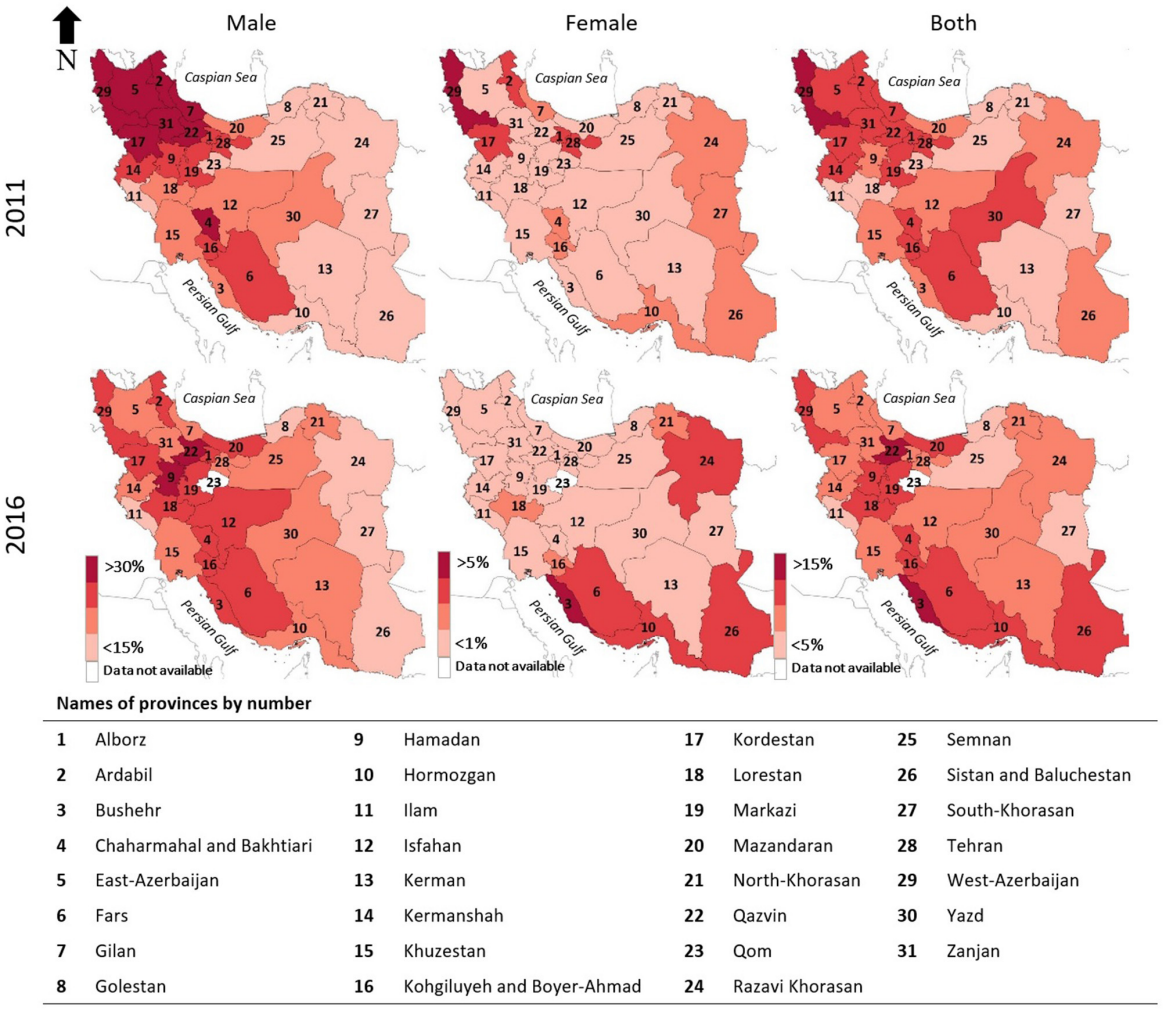
Geographical pattern of current tobacco smoking in Iran by province in 2011 and 2016.

There are 31 provinces in Iran. The current tobacco smoking prevalence showed a geographical pattern throughout the country, which was more distinct among men in 2011. In 2011, the highest current tobacco smoking prevalence nationwide was among those who lived in north-western provinces, including West-Azerbaijan, East-Azerbaijan, Ardabil, Kordestan, Zanjan, Qazvin, and Gilan. In 2016, men in Hamadan and Qazvin had the highest current tobacco smoking prevalence. The current tobacco smoking prevalence among men had a gradient from west to east with western provinces having higher prevalence. Among women, the current tobacco smoking prevalence in 2011 was highest in West-Azerbaijan, in the north-west, Razavi-Khorasan and South-Khorasan in the northern east and east, and Hormozgan, in the south. However, in 2016, women who lived in Bushehr, located in the south-west, Fars and Hormozgan, in the south, Sistan and Baluchestan in the south-east, and Razavi-Khorasan in the north-east had the highest current tobacco smoking prevalence (Figure 1).

### Time Trend of Current Tobacco Smoking Prevalence During 2004-2016

**Figure 2:**
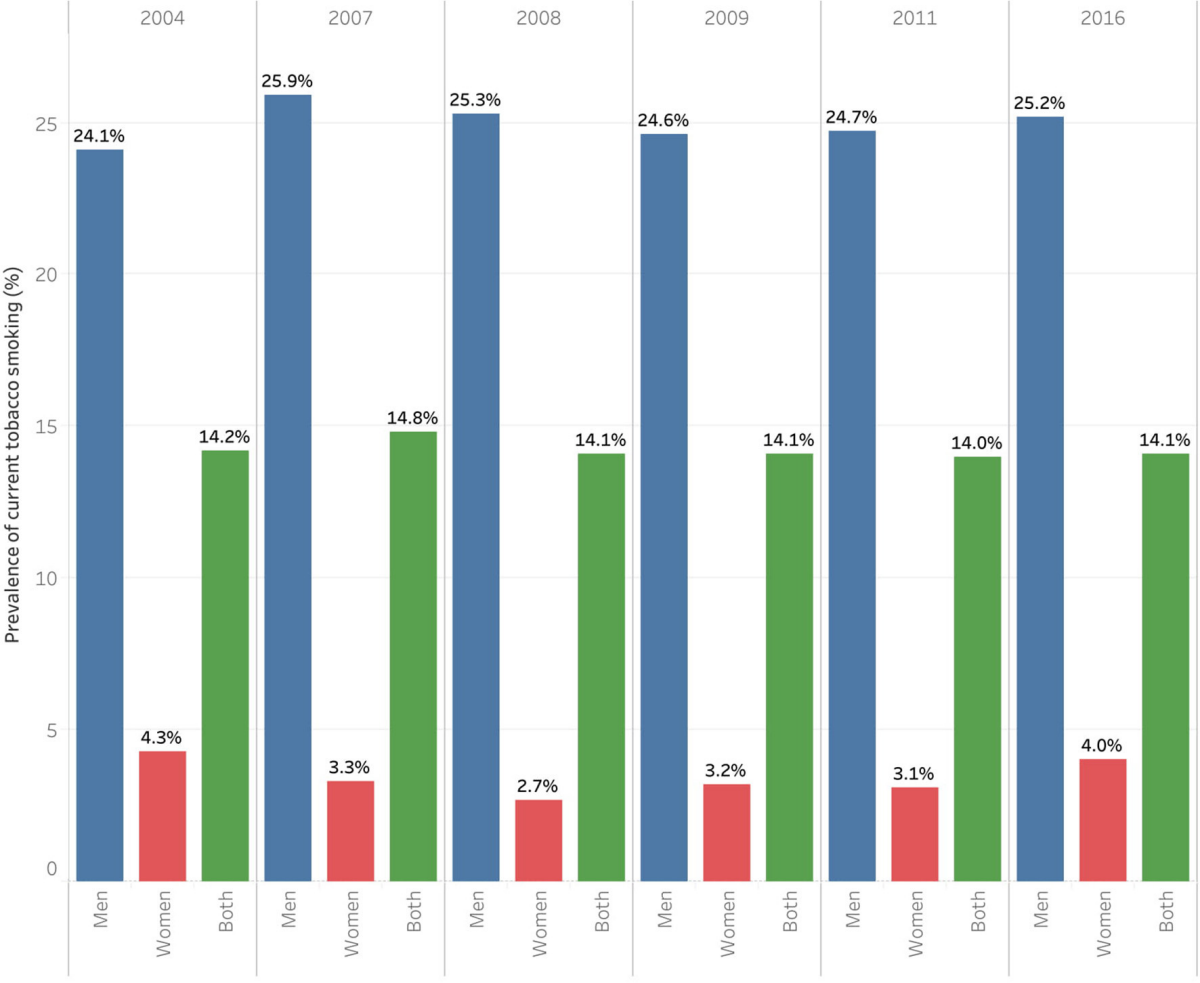
Prevalence of current tobacco smoking among women and men in 2004, 2007, 2008, 2009, 2011, and 2016.

The current tobacco smoking prevalence among Iranian adults remained unchanged during 2004-2016 among both men and women, *p* > 0.05. In addition, the current tobacco smoking prevalence has remained significantly higher among men than that of women, *p* < 0.001 (Figure 2).

## Discussion

The study showed that 14.1% of Iranian adults in 2016 were current-tobacco-users. The current tobacco smoking prevalence was 25.2% among men and 4.0% among women. Worldwide, the prevalence of tobacco smoking among women is less than men (10). The current tobacco smoking prevalence in Iran was lower than most countries in the region (11): Armenia 26.9%, 49.9% among men and 1.6% women (12); Turkey 31.5%, 43.4% men 19.7% women (13); Egypt 22.7%, 43.4% men and 0.5% women (14); Iraq 19.6%, 36.1% men and 1.8% women (15); Azerbaijan 35.3% men and zero among women (16); and Bahrain 17.9%, 30.6% men and 5.7% women (17). All mentioned neighboring countries are located northern and western of Iran except for Egypt and Bahrain. The current tobacco smoking prevalence in Iran was higher than Afghanistan 8%: 15% men and 0.2% women (18); Pakistan 12.7%, 25.5% men and 3.8% women (19); and Qatar 12.1%, 20.2% men and 3.1% women (20). Afghanistan and Pakistan are the eastern neighboring countries of Iran. In contrast to Iran where chewing tobacco is very unpopular, the prevalence of chewing tobacco in Afghanistan was 25.4% among men and 0.6% among women (18). Tobacco use has been associated with factors including socioeconomic status, education, age, employment, income, marital status, psychological factors, and religious beliefs (21). All the mentioned factors vary significantly among the countries in the region.

In 2011, the highest current tobacco smoking prevalence nationwide was among men who lived in the north-western provinces. The geographical pattern in 2016 among men was changed and the current tobacco smoking prevalence was highest in the central-west. However, it could be suggested that in both 2011 and 2016, the current tobacco smoking prevalence was higher among men who lived in western provinces in Iran, especially the north-west, than those who lived in eastern provinces. The geographical pattern from 2011 to 2016 among women also changed. In 2011, the current tobacco smoking prevalence among women was highest in the north-west, the east, and the south. However, in 2016 provinces in south-west, south-east, and north-east were added to provinces which had the highest current tobacco smoking prevalence among women.

The geographical distribution of current tobacco smoking prevalence among neighboring countries conformed to that of the provinces in Iran. The ethnicity of people has probably given rise to similarities in culture and behavior. Among provinces in the north-west and central-west, the people who live in West-Azerbaijan, East-Azerbaijan, Ardabil, Zanjan, and to some extent Qazvin share similarities with the neighboring countries Turkey (13, 22) and Azerbaijan (16) which could have been reflected in their smoking behavior. Similarly, the smoking behavior of people in Kordestan province in Iran, the majority of whom were of Kurd ethnicity, was similar to that of people who lived in the Kurdistan region in Iraq (23, 24). In addition, the smoking behavior of people in eastern provinces of Iran was similar to that of eastern neighboring countries including Afghanistan and Pakistan (18, 19).

The mean number of daily smoked cigarettes was 14.6 among men and 13.1 women, which was much lower than that of Armenia where it was 22.7: 22.9 among men and 15.6 women (12). However, it was higher than Pakistan with 9.4: 10.7 among men and 2.9 women (19). The average number of cigarettes smoked per day among daily cigarette smokers in Egypt was 15.5: 15.9 among men and 12.3 women (14); in Iraq 15.5: 15.9 among men and 12.3 women (15); and in Turkey 15.5 (13): 16.8 among men and 12.7 women.

Only 0.2%, 0.3% among men and 0.1% women, of participants reported smoking water-pipe. This was much lower than the prevalence of current-water-pipe-smokers in Bahrain: 8.4% (17), Egypt: 4.5% (14), or Iraq: 1.7% (15). Similar to Iran, water-pipe use in Egypt was more prevalent among men than women: 8.7% vs. 0.1% (14). In addition, only men reported to use water-pipe in Iraq (15).

Water-pipe use is considered recreational in eyes on public in Iran. Setting up water-pipe is time and money consuming and also needs special equipment. Although there is a public ban against water-pipe use, the recreational use of water-pipe does not usually result in legal consequences.

The mean onset age of smoking was 21.4 among men and 27.8 among women. The mean onset age of smoking among countries in the region was higher in Iraq: 23.9 years among men and 19.2 women (15); lower in Turkey: 17.2 years men and 20.2 women (13); lower in Armenia: 17.9 men and 26.2 women (12); and same in Pakistan: 21.5 men and 24.9 women in Pakistan (19).

Among all tobacco smokers, only 14.4% had attempted to quit smoking during the previous year: 25.5% among men and 12.4% among women. This figure is much lower than Egypt, 40.3% (14), and Turkey, 27.4% (13). More than half, 57.2%, of participants in Iraq reported they had tried to quit smoking (15).

Some 35.5% of smokers who had seen a healthcare professional during the previous year had been advised to quit smoking. This is higher than Egypt (14) and Turkey (13) where 28.4% and 22.3% of smokers had been advised by a healthcare provider to stop smoking in the previous year. Smokers who had been advised to quit smoking were more likely to attempt quitting smoking compared with those who hadn't. Therefore, more attention need to be paid to the unhealthy lifestyle of patients who visit doctors, and follow-up programs be established to help those smokers quit smoking.

The prevalence of passive smoking at home or workplace was 27.4%. The prevalence of passive smoking in Egypt was higher: 48.9% at home and 30.3% at workplace (14). In addition, half of respondents in Iraq were passive smokers at home (52.8%) and workplace (56.1%) which was higher than Iran (15).

The prevalence of passive smoking in Iran was not significantly different among men and women; however, it was higher among men than women in Iraq (15). While women were more likely to be passive smokers at home, men were more likely to be passive smokers at work. The proportion of passive smokers at work was higher in urban than rural areas. The majority of women were housewives and therefore could have been passive smokers if their husbands smoked. The majority of people in rural areas live in houses with single rooms. In addition, women in the rural areas are more likely to consider smoking a right of men in their family which puts them at risk of passive smoking.

The current tobacco smoking prevalence among Iranian adults remained unchanged during 2004-2016 for both men and women. Iran verified the WHO Framework Convention on Tobacco Control (FCTC) in 2003. The treaty came into force in February 2005 by WHO, and in October 2005 Iran's Comprehensive National Tobacco Control Act was approved (25, 26). The national committee of control and combating tobacco was then established, whose task included codification of executive orders related to definitions and advertising features, drafting and adoption of training programs and researches in collaboration with related organizations, and editing of messages, warnings, images, and designs associated with the social, economic and health adverse effects of tobacco and their time bounding (27). The use of any smoked tobacco product in public places were forbidden (28). In addition, smoking cessation counseling services were to be provided in all health centers for people who were willing to quit smoking. Warning statements about dangers of tobacco on any tobacco product was known mandatory, and distribution of tobacco products with no health warning labels was considered illegal. In addition, the use of misleading marketing terms such as light and low-coal-tar on the packaging was prohibited. During the annual national anti-smoking week, 3rd to 10th of June, public campaigns and training sessions would be held to increase awareness about adverse effects of smoking while also informing people about the control and anti-smoking programs (29). Health centers would provide training sessions for people and particularly target groups of tobacco control program including students, the youth, wife of smokers and smokers themselves.

Various policies have been implemented worldwide to reduce the prevalence of tobacco-use, including smoking bans, increasing tax, training, and counseling. Although smoking bans have been introduced to reduce smoking-prevalence and increase quit ratio, their actual impact on smoking cessation remains unclear. Smoking ban implementation resulted in a temporary reduction of smoking-prevalence and increase in quit ratio; however, the rates progressively returned to the levels prior to smoking ban (30). Therefore, it seems that smoking bans cannot solely be effective in reducing smoking-prevalence. England is a leading European country in the implementation of tobacco control programs (31). They managed to reduce the smoking-prevalence from 27% in 2000 to 16% in 2016 in absolute terms, 0.72% annual reduction (32). A study reported that 15% of the reduction could be attributable to the Stop Smoking Services, approved by the English National Health Service (33). Smokers who were motivated to quit were referred to Stop Smoking Services. They would participate in special training courses. In addition, evidence-based behavioral and pharmacological interventions would be provided by trained healthcare professionals for smoking cessation including cessation specialists or trained general practitioners, nurses, pharmacists or dentists. An advantage of Stop Smoking Services in comparison with other quit attempts was that Stop Smoking Services combined pharmacological interventions with behavioral counseling. Although counseling and follow-up could be provided by cessation specialists or other trained healthcare professionals, cessation specialists were more successful in preventing smoking relapse. However, specialist cessation support may not be necessary to all quit attempts, and it could be suggested that quit programs could be a mix of specialist support for a small proportion of smokers who need it, brief support along with cessation medication for many and self-help for the majority (33).

Although studies suggest that increased tax of tobacco products could result in the reduction of its consumption, it has remained challenging for authorities. Wide boundaries with neighboring countries which facilitates the trafficking of cheap tobacco products is probably the main reason to avoid tax increases.

Finally, it seems that measures taken so far have not been effective in decreasing the current tobacco smoking prevalence in Iran. It could be suggested that more strict legislations be provided by the parliament and the government to discourage smoking. In addition, more innovative solutions need to be employed to help smokers quit smoking.

## Conclusion

The current tobacco smoking prevalence among Iranian population has not changed significantly during 2004-2016 and does not conform to the international guidelines. Therefore, it remains crucial yet challenging that effective nationwide policies be implemented to reduce the use of tobacco products. One cannot hope for any reductions in smoking prevalence until a cocktail of interventions are built around strong commitment to government policy.

## Data Availability Statement

The datasets presented in this article are not readily available because: The authors need to grant permission of the National Institute of Health of Iran. Requests to access the datasets should be directed to Ali-Asghar Kolahi, a.kolahi@sbmu.ac.ir.

## Ethics Statement

The study was approved by Ethical Committee of Shahid Beheshti University of Medical Sciences under the reference code IR.SBMU.RETECH.REC.1397.068. As stated in the protocol of STEPS surveys, participants provided their written consent prior to taking part in the study.

## Author Contributions

A-AK, M-RS, and MA-K: conceptualization and data collection. MA-K: writing—original draft. A-AK and M-RS: writing—review & editing. A-AK: resources and supervision. All authors contributed to the article and approved the submitted version.

## Conflict of Interest

The authors declare that the research was conducted in the absence of any commercial or financial relationships that could be construed as a potential conflict of interest.
